# An Information-Theoretic Machine Learning Approach to Expression QTL Analysis

**DOI:** 10.1371/journal.pone.0067899

**Published:** 2013-06-25

**Authors:** Tao Huang, Yu-Dong Cai

**Affiliations:** 1 Institute of Systems Biology, Shanghai University, Shanghai, P. R. China; 2 Department of Genetics and Genomic Sciences, Mount Sinai School of Medicine, New York, New York, United States of America; University of California, Riverside, United States of America

## Abstract

Expression Quantitative Trait Locus (eQTL) analysis is a powerful tool to study the biological mechanisms linking the genotype with gene expression. Such analyses can identify genomic locations where genotypic variants influence the expression of genes, both in close proximity to the variant (cis-eQTL), and on other chromosomes (trans-eQTL). Many traditional eQTL methods are based on a linear regression model. In this study, we propose a novel method by which to identify eQTL associations with information theory and machine learning approaches. Mutual Information (MI) is used to describe the association between genetic marker and gene expression. MI can detect both linear and non-linear associations. What’s more, it can capture the heterogeneity of the population. Advanced feature selection methods, Maximum Relevance Minimum Redundancy (mRMR) and Incremental Feature Selection (IFS), were applied to optimize the selection of the affected genes by the genetic marker. When we applied our method to a study of apoE-deficient mice, it was found that the cis-acting eQTLs are stronger than trans-acting eQTLs but there are more trans-acting eQTLs than cis-acting eQTLs. We compared our results (mRMR.eQTL) with R/qtl, and MatrixEQTL (modelLINEAR and modelANOVA). In female mice, 67.9% of mRMR.eQTL results can be confirmed by at least two other methods while only 14.4% of R/qtl result can be confirmed by at least two other methods. In male mice, 74.1% of mRMR.eQTL results can be confirmed by at least two other methods while only 18.2% of R/qtl result can be confirmed by at least two other methods. Our methods provide a new way to identify the association between genetic markers and gene expression. Our software is available from supporting information.

## Introduction

As a powerful tool to increase understanding of the biological mechanisms by integrating genetic marker data with gene expression data [1], the goal of expression Quantitative Trait Locus (eQTL) analysis is to identify genomic locations where genotype significantly affects gene expression [2]. This analysis was first applied to yeast [3] and then to mouse and human [4]. Many cis/trans loci associated with the expression level of hundreds of transcripts were identified. In cis-acting eQTLs, the SNPs are close to the affected gene; while in trans-acting eQTLs, the SNPs are far away from the affected gene. Usually, the trans-effects are weaker than the cis-effects, but the number of trans-effects is larger than the cis-effects in mouse and human [2], [4]. How close the SNP and the affected gene should be in cis-acting eQTLs is debatable [2]. In this study, the SNPs that were within 5 Mb of the affected genes [5] were termed cis-acting eQTLs.

Since eQTL is intended to assist in discovery of whether the genetic marker at a certain locus is correlated with the gene expression of a certain gene, the traditional eQTL methods are based on the linear regression of the gene expression with the genetic marker [6]. The expression level of one gene is assumed to be the result of one or multiple genetic markers [7]. But on the other hand, we can also say that one genetic marker can affect one or multiple genes. The relationship between genetic marker and gene expression is mutual.

Unlike traditional statistical eQTL methods, here we propose an information theory based machine learning method to accomplish eQTL analysis. It is different from traditional statistical eQTL methods in the following ways:

First, the association between genetic marker and gene expression is measured with Mutual Information (MI), which can not only be used for both linear and non-linear dependencies, but can also capture the potential heterogeneity of the study population [8]. As an ideal stochastic dependence measurement [9], MI considers all types of dependencies, including linear relationships and monotonic dependencies [10]. MI measures the mutual dependence between two variables [11]–[13]. The MI between X and Y is defined as the marginal entropies of X minus the conditional entropies of X|Y. The marginal entropies of X measure the uncertainty of variable X. The conditional entropies of X|Y measure the uncertainty remaining about X after Y is given. Since MI is symmetric [14], i.e., the MI of X and Y is the same as the MI of Y and X, the MI between X and Y equals the marginal entropies of Y minus the conditional entropies of Y|X as well.

Second, in this method, the status of the genetic marker is considered the class label and the expression levels of genes are considered features. The expression levels of genes are then used to predict the status of the genetic marker. The idea of predicting genotype from gene expression is originated from a series of reverse engineering works from gene expression to its genetic basis [15]–[17]. The genes that one genetic marker can affect are determined by both the MI between the gene and the genetic marker, and the MI among genes. In other words, not only the relevance between the gene and the genetic marker, but also the redundancy among genes was considered. The relevance guarantees detection of the strong associations and the redundancy can filter the indirect associations. The affected genes are optimized with feature selection techniques: Maximum Relevance Minimum Redundancy (mRMR) and Incremental Feature Selection (IFS). The biological rationale of applying these feature selection method to eQTL study is that if a set of genes are highly co-expressed, they are very likely involved in the same biological process and have similar biological functions. The feature selection procedure will reduce the number of regulated genes but the representative ones will be selected. The biological functions of regulated genes by the SNP will be clearer.

Our methods provide a new way to identify the associated genetic marker - gene expression eQTL pairs with advanced information theory and optimize the detection of affected genes corresponding to genetic markers with feature selection and machine learning. We applied the method on a published eQTL mouse data set [18] and simulated data set. On the published data set, our method identified more consensus eQTLs than traditional methods. On the simulated data set, the area under the precision-recall curve (AUPR) of our method is greater than traditional methods, such as R/qtl [19], and MatrixEQTL (modelLINEAR and modelANOVA) [20].

## Materials and Methods

### Dataset

The eQTL dataset we used were obtained from a published study by Jonathan David Smith [18]. The gene expression data was downloaded from Gene Expression Omnibus (GEO) with accession number of GSE8512. The SNP data was provided by Jonathan David Smith [18]. There were 207 apoE-deficient F2 mice utilized from an AKRxDBA/2 intercross. The numbers of male and female mice were 114 and 93, respectively. The gene expression was measured with Affymetrix Mouse Genome 430 2.0 Array. There were 45,101 probes in this platform. Meanwhile, the genotypes of 1,967 informative SNP markers: 1 = homozygous AKR allele, 2 = heterozygous, 3 = homozygous DBA/2 allele, were measured.

The genotype of SNP 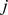 can be inferred based on genes regulated by SNP 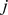


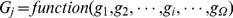
(1)


Where 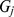 is the genotype of SNP 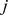, 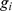 is the expression level of regulated gene 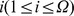 and,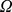 depends on the selection of regulated genes which will be elaborated below.

In the original study by Jonathan David Smith [18], the R/qtl [19] software was used to detect the eQTLs. In their eQTL analysis, the association between phenotype and locus was determined by linear correlation analysis using both dominant and additive models, and the model with the highest correlation coefficient was selected [18], [21].

### mRMR Method

We used the mRMR method [22], [23] to rank the genes according both to their relevance to the genotype and to the redundancy among the genes. The genes with top ranks should have maximum relevance to the genotype class and also be minimally redundant, i.e., maximally dissimilar to one another. The maximum relevance makes sure that the genes are associated with the genotype and the minimum redundancy reduces the indirect associations. Both relevance and redundancy are defined by mutual information (MI), which measures how much one vector is related to another. MI is defined as follows:
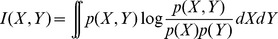
(2)where 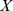 and 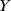 are two vectors, 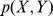 is the joint probabilistic density, 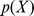 and 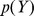 are the marginal probabilistic densities.

Let 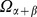 denotes the whole vector set containing all the genes, 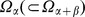 denotes the selected vector set with 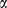 vectors, and 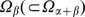 denotes the to-be-selected vector set with 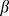 vectors. The relevance 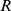 of a gene 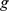 in 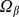 with the genotype variable 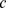 can be computed by equation (3):
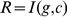
(3)


The redundancy 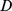 of a gene 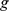 in 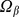 with all the genes in 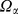 can be computed by equation (4):
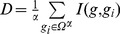
(4)


To obtain a gene 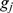 in 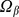 with maximum relevance and minimum redundancy, the mRMR function is obtained by integrating equation (3) and equation (4):
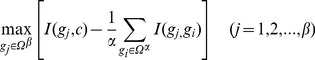
(5)


For a gene pool containing 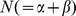 genes, the evaluation will be executed in 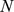 rounds. After these evaluations, an ordered gene set 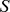 will be obtained:
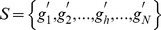
(6)where each gene in 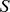 has a subscript index, indicating at which round the gene is selected. The better a gene is, the earlier it will satisfy equation (5) and be selected, and the smaller its subscript index will be.

The mRMR software we used was downloaded from http://penglab.janelia.org/proj/mRMR/.

If there are covariates that should be adjusted, the conditional mutual information can be used to replace mutual information in equation (5). The modified equation is equation (7)
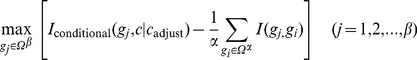
(7)where 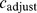 are the covariates that should be adjusted, there can be several covariates; 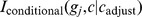 is the conditional mutual information between 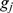 and 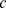 with adjustment of 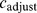. It can be calculated by equation (8)
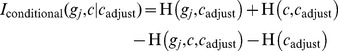
(8)where H is the entropy of the empirical probability distribution.

### Nearest Neighbor Algorithm

In our work, the Nearest Neighbor Algorithm (NNA) [24]–[28] was used to classify mice into different genotypes. The basic idea is to assign a new mouse to its genotype by comparing the genes of this mouse with the genes of those that have known genotypes. The distance between two mice 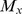 and 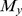 in the study is defined as [24]–[28]:
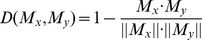
(9)where 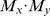 is the inner product of 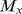 and 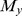, and 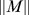 is the Euclidean norm of vector 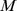. The smaller 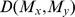 is, the similar 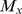 and 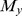 are.

In NNA, a vector 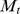 will be designated as having the same class as its nearest neighbor 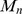 which has the smallest 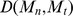. That is
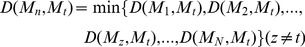
(10)where 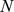 represents the number of training mice.

### Jackknife Cross-Validation Method

The Jackknife Cross-Validation Method, also known as Leave-One-Out Cross-Validation (LOOCV), is one of the most effective and objective ways to evaluate statistical predictions [29]–[31]. In the Jackknife Cross-Validation Method, each sample in the dataset is knocked out in turn and tested by the predictor, which is trained by the other samples in the data set [25], [28], [32]–[39]. During this process, each sample is involved in training 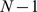 times and is tested exactly once. To evaluate the performance of the predictor, the accuracy rate for the overall samples can be calculated as:
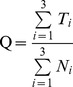
(11)where 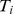 and 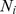 stand for the number of correctly predicted mice and overall mice in genotype 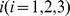. Genotype 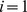 means homozygous AKR allele, 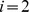 means heterozygous, 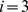 means homozygous DBA/2 allele.

The accuracy of genotype prediction evaluated by Jackknife Cross-Validation was used as a measurement of explanation ability of gene set expression to SNP.

### Incremental Feature Selection (IFS)

After the mRMR step, we obtained a gene list in their order of selection. However, we still do not know how many genes in the list should be chosen. In our study, Incremental Feature Selection (IFS) [25], [28], [32]–[38] was used to determine the optimal number of genes. We constructed 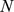 gene subsets of the gene list 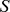 provided by the mRMR gene list defined in equation (6) by adding an additional gene to the candidate gene subset, starting from an initial subset containing only the first gene 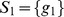. The gene subset 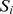 is defined as:
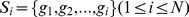
(12)by adding gene 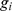 to the previous subset 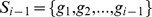.

For each gene subset 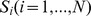, the Jackknife Cross-Validation Method is used to obtain the accuracy rate. The results were plotted to produce an IFS curve with index 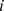 as its x-axis and the overall accuracy as its y-axis. The optimal genes were defined as the genes that reach the highest accuracy.

### The Workflow of mRMR.eQTL

**Figure 1:**
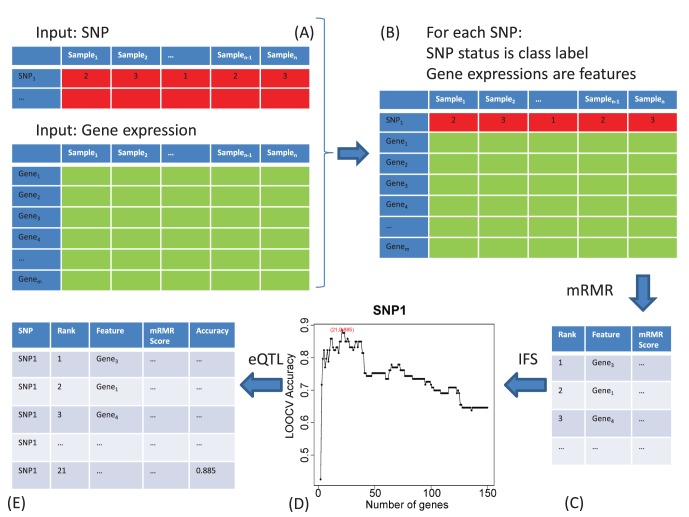
The workflow of mRMR.eQTL. (A) The input of mRMR.eQTL includes genotype and gene expression data of the same samples. (B) For each SNP, the SNP status is considered as class label and the gene expressions are considered as features. (C) mRMR feature selection is applied to rank the genes based on its relevance to the genotype and redundant to other genes. (D) Incremental feature selection is applied to select the optimal gene set that can best discriminate the genotype status. (E) The eQTL tables are generated based on the mRMR and IFS results.

A pipeline of above analysis procedures were illustrated in **Figure 1**. The software that implements this pipeline is called mRMR.eQTL. There are five steps:

First, the input of mRMR.eQTL includes genotype and gene expression data of the same samples.

Second, for each SNP, the SNP status is considered as class label and the gene expressions are considered as features.

Third, mRMR feature selection is applied to rank the genes based on its relevance to the genotype and redundant to other genes. The feature selection will generate two lists: the mRMR and MaxRel list. The MaxRel list is ranked based on relevance. The mRMR list is ranked based on relevance and redundancy. The user can choose use which list in the IFS.

Fourth, IFS is applied to select the optimal gene set that can best discriminate the genotype status.

Fifth, the eQTL tables are generated based on the mRMR and IFS results.

The mRMR.eQTL software is available in **Script S1**.

## Results and Discussion

### mRMR Results

Since the mice we studied were apoE-deficient F2 mice from an AKRxDBA/2 intercross, for each genetic marker, i.e. SNP, there were three statuses: homozygous AKR allele, heterozygous allele and homozygous DBA/2 allele. The genotype is the category variable which is usually the target class label in machine learning studies. The gene expression level is the numeric variables which contains data that can be used as features to represent the target class label. Therefore, unlike traditional eQTL methods which use the genotype data to represent the expression data, we used the expression data as features to represent the genotype data which were target class labels. After transforming the eQTL problems, i.e. discovering correlations between genotype data and expression data and placing these into machine learning questions, we applied the advanced mRMR methods to extract informative genes that have maximal relevance to the genotype and at the same time have minimal redundancy among genes. The maximal relevance ensures that the selected genes were strongly correlated with the genotype. The minimal redundancy reduces the number of selected genes; such a compact gene set will have fewer false positive correlations.

The genotype of each genetic marker was considered a machine learning problem. The genes were ranked by relevancy with the corresponding genetic marker. After the mRMR analysis, we obtained the mRMR score and mRMR order of all genes for each genetic marker. The mRMR score can be used a measurement of association between gene expression and genetic marker.

### IFS Results

With mRMR analysis, we can obtain the rank of association between gene expression and genetic marker, but it is still not clear how many genes are affected by the SNP. The number of affected genes can be optimized with IFS methods. In this method, the genes were progressively tested and the gene set that achieves the best prediction performance is considered the optimal gene set. Unlike the traditional eQTL methods which usually require an arbitrary cutoff, the IFS method is parameter free. It utilizes the IFS curve which characterizes the distribution of prediction performances, to optimize the affected gene selection.

### eQTL Results

After the feature selection with mRMR and IFS, we obtained 6489 and 9401 eQTLs in female and male mice, respectively.

If the distance between SNP and the affected genes was smaller than 5 Mb [5], this eQTL association was termed a cis-acting eQTL. Since the sequences of some probes in the Affymetrix Mouse Genome 430 2.0 Array did not achieve a perfect match with the mouse genome, they did not have exact genome locations. If an eQTL pair includes such probes, it was then termed ambiguous eQTL. Based on the above criteria, there were 1298 cis-acting eQTLs, 3392 trans-acting eQTLs, 1799 ambiguous eQTLs in female mice and 1698 cis-acting eQTLs, 5324 trans-acting eQTLs, 2379 ambiguous eQTLs in male mice.

To investigate the differences between cis-acting eQTLs and trans-acting eQTLs, we compared the mRMR scores of cis-acting eQTLs and trans-acting eQTLs and found that the mRMR scores of cis-acting eQTLs were significantly greater than the mRMR scores of trans-acting eQTLs. The one sided t test p values of this comparison in female and male mice were 7.13e-47 and 1.43e-66, respectively. Although the trans-acting eQTLs were weaker than cis-acting eQTLs, the number of trans-acting eQTLs was larger than the number of cis-acting eQTLs. Both the comparisons of associations and numbers of cis-acting eQTLs and trans-acting eQTLs in our results agreed with the prior reports that in mouse, the cis-acting eQTLs are stronger than trans-acting eQTLs but that there are more trans-acting eQTLs than cis-acting eQTLs [2].

### Comparison with the Original eQTL Results

**Figure 2:**
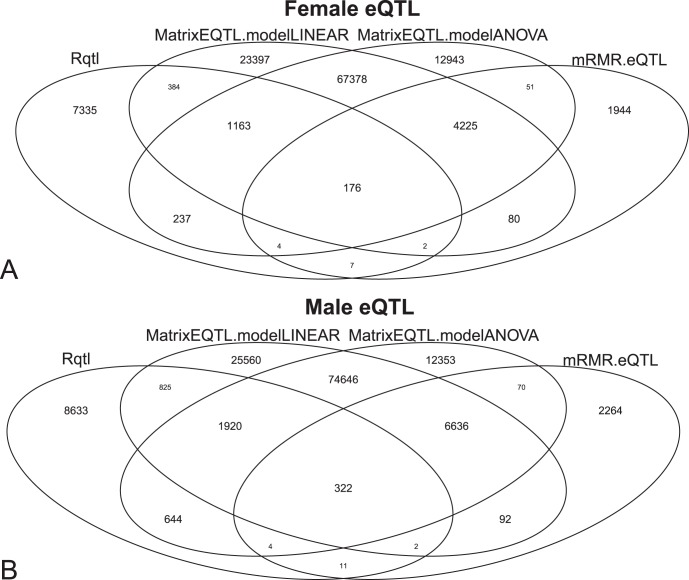
The venn diagram of mRMR.eQTL, R/qtl, MatrixEQTL.modelLINEAR and MatrixEQTL.modelANOVA in female and male mice. (A) The venn diagram of mRMR.eQTL, R/qtl, MatrixEQTL.modelLINEAR and MatrixEQTL.modelANOVA in female mice; (B) The venn diagram of mRMR.eQTL, R/qtl, MatrixEQTL.modelLINEAR and MatrixEQTL.modelANOVA in male mice.

We compared our eQTL results with Jonathan David Smith’s results [18]. They used R/qtl to calculate the eQTLs in female and male mice. Recently, a new method of eQTL, MatrixEQTL [20], was developed. MatrixEQTL includes two models: modelLINEAR and modelANOVA. In modelLINEAR, the effect of genotype is considered as additive linear and the significance is tested using t-statistic. In modelANOVA, the genotype is treated as categorical variable and ANOVA model is applied to test the significance. We calculated the eQTLs in female and male mice using MatrixEQTL.modelLINEAR and MatrixEQTL.modelANOVA as well. **Figure 2** shows the venn diagram of these four methods. The cutoff of R/qtl was log-odds (LOD) > = 3. The cutoff of MatrixEQTL.modelLINEAR and MatrixEQTL.modelANOVA was False Discovery Rate (FDR) < = 0.05. The cutoff of mRMR is LOOCV Accuracy> = 0.90. The outputs of these four methods were given in **Dataset S1**. In female mice, 67.9% of mRMR.eQTL results can be confirmed by at least two other methods while only 14.4% of R/qtl result can be confirmed by at least two other methods. In male mice, 74.1% of mRMR.eQTL results can be confirmed by at least two other methods while only 18.2% of R/qtl result can be confirmed by at least two other methods. Our method, mRMR.eQTL was better than R/qtl, which was used in the original study by Jonathan David Smith [18].

### Biological Relevance of the eQTL Results

The goal of eQTL analysis is to discover associations between genetic markers which mark the genome locations and genes whose expression level are affected by the genetic markers. Such SNP - gene associations can enhance the understanding of biological mechanisms. Here, the mice we studied were apoE-deficient F2 mice from an AKRxDBA/2 intercross.

To investigate the roles of Apoe in the eQTL associations, we extracted its interaction partners from STRING (http://string-db.org/) [40]. STRING is a comprehensive and widely used [32], [33], [41]–[44] protein interaction database. Since each SNP corresponding to some affected genes, we sought to find which SNPs have significantly more interaction partners of Apoe. We did an hypergeometric test [32]–[34], [36]–[38] to analyse the overlap between Apoe’s partners and affected genes by each SNP and found the SNPs that have significantly more than random Apoe partners with a hypergeometric test p value less than 0.05. The enriched SNPs in female and male mice are given in **Table 1** and **Table 2**, respectively.

**Table 1:** SNPs with significantly more Apoe partners in female mice.

SNP	Gene located close to the SNP	P value	Number of Apoe partners	Apoe partners
rs6350987	Kcna4	0.003421984	3	Cat, Cd44, Rbm45
rs13476656	Gm13803	0.005736386	3	Cat, Cd44, Rbm45
rs13476672	Cd44	0.005736386	3	Cat, Cd44, Rbm45
rs3689502	Gm13803	0.005736386	3	Cat, Cd44, Rbm45
rs6246565	Hsd17b12	0.005736386	3	Cat, Cd44, Rbm45
rs13478827	Gm8992	0.030289618	2	Gpnmb, Apobec1

**Table 2:** SNPs with significantly more Apoe partners in male mice.

SNP	Gene located close to the SNP	P value	Number of Apoe partners	Apoe partners
rs13480712	Hal	0.007091998	12	Ebp, Npc2, Pla2g2e, Lta4h, Vapb, Enpp1, Irak1, Ncor2, Gla, Ccl24, Cbx3, Hecw1
rs13481811	BB123696	0.007278187	3	2010111I01Rik, Sptlc1, Nrip1
rs13480667	Ikbip	0.008178776	9	Npc2, Ngb, Pla2g2e, Lipg, Lta4h, Enpp1, Tax1bp1, Nr0b2, Il6st
rs13481820	Gm19516	0.011111922	3	Stab2, 2010111I01Rik, Sptlc1
rs13481821	Slc25a48	0.011111922	3	Stab2, 2010111I01Rik, Sptlc1
rs3698807	Gm19516	0.011111922	3	Stab2, 2010111I01Rik, Sptlc1
rs8273881	Slc34a1	0.011111922	3	Stab2, 2010111I01Rik, Sptlc1
rs13480704	Mir135a-2	0.014548555	7	Npc2, Pla2g2e, Lipg, Lta4h, Vapb, Enpp1, Il6st
rs13480695	Nr1h4	0.014572157	11	Plat, Npc2, Pla2g2e, Lipg, Usp12, Lta4h, Enpp1, Plek, Nr0b2, Apaf1, Il6st
rs13481896	LOC101055640	0.015906918	3	Stab2, 2010111I01Rik, Sptlc1
rs13478738	Cntnap2	0.019373037	4	Dfna5, Armc9, Pnlip, Gpnmb
rs13481850	Ercc6l2	0.021689728	3	Stab2, 2010111I01Rik, Sptlc1
rs3705446	Arrdc3	0.021689728	3	Stab2, 2010111I01Rik, Sptlc1

**Figure 3:**
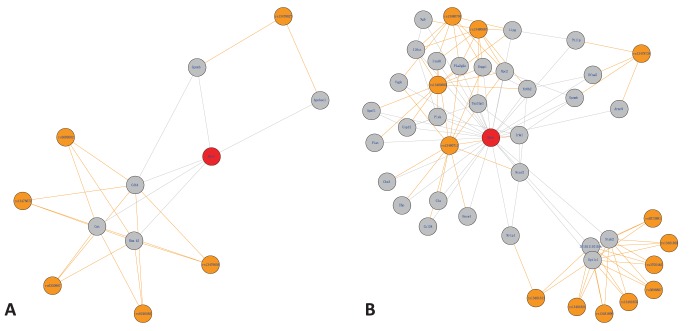
The network of Apoe partners and their upstream SNPs. (A) The network of Apoe partners and their upstream SNPs in male mice. The red node is Apoe. The grey nodes are Apoe partners. The orange nodes are their upstream SNPs. The grey edges are protein-protein interactions. The orange edges are eQTL relationships between SNPs and genes. (B) The network of Apoe partners and their upstream SNPs in female mice. The red node is Apoe. The grey nodes are Apoe partners. The orange nodes are their upstream SNPs. The grey edges are protein-protein interactions. The orange edges are eQTL relationships between SNPs and genes.

To view the manner in which the SNPs affect their downstream genes and in which these genes interact with Apoe, we plotted **Figure 3** which shows the eQTL associations between the enriched SNPs and their downstream genes in female and male mice and the protein interactions between Apoe and its partners. In female mice, two SNPs of Gm13803, rs13476656 and rs3689502, regulates three Apoe’s interaction partners, Cat, Cd44 and Rbm45. In male mice, two SNPs of Gm19516, rs13481820 and rs3698807, regulates three Apoe’s interaction partners, Stab2, 2010111I01Rik and Sptlc1. The eQTL results provided useful clues about the functions of predicted genes, Gm13803 and Gm19516.

### Comparison with other Methods on Simulated Dataset

**Figure 4:**
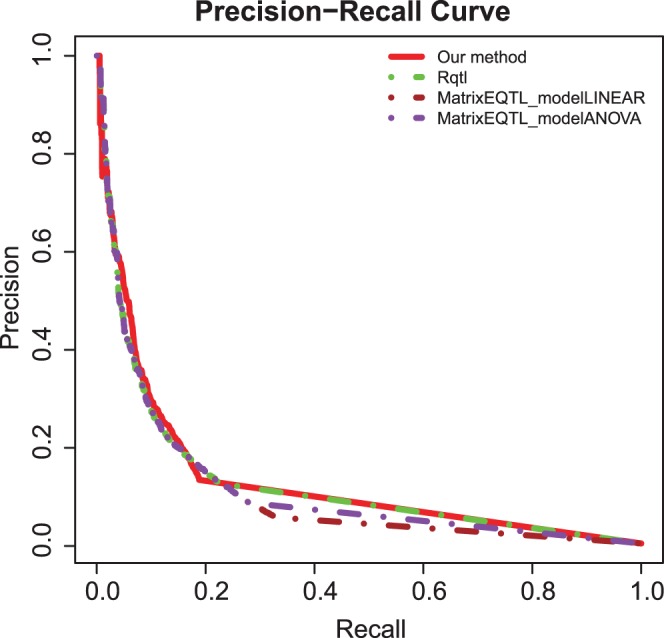
The precision-recall curves of our method, R/qtl, and MatrixEQTL (modelLINEAR and modelANOVA). The red, green, brown, purple lines represent the precision-recall curves of our method, R/qtl, MatrixEQTL_modelLINEAR and MatrixEQTL_modelANOVA, respectively.

We generated simulated genotype and gene expression data using SysGenSIM [45]. The parameters used in the simulation are as following: population size with 250, size of genes/SNPs with1000, network topology with small-world, and average degree of node with 10. To evaluate the eQTL identification performance of our method, R/qtl, and MatrixEQTL (modelLINEAR and modelANOVA), we plotted the precision-recall curve and calculate the area under the precision-recall curve (AUPR) which was widely used in evaluating eQTL and network construction methods [46]–[48]. For calculating the precision-recall, the MaxRel score in our method was used as a measurement to get the eQTL prediction. In R/qtl, the prediction measurement is LOD. For MatrixEQTL (modelLINEAR and modelANOVA), the prediction measurement is 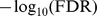. The precision-recall curves of our method, R/qtl, and MatrixEQTL (modelLINEAR and modelANOVA) were shown in **Figure 4**.

The AUPR and relative AUPR (RAUPR) of our method, R/qtl, and MatrixEQTL modelLINEAR and MatrixEQTL modelANOVA were given in **Table 3**. The RAUPR scaled the AUPR to the maximum value obtained across the four methods [49]. Our method has the greatest AUPR among the four methods.

**Table 3:** The AUPR comparison of our method, R/qtl, and MatrixEQTL modelLINEAR and MatrixEQTL modelANOVA.

	Our method	R/qtl	MatrixEQTL modelLINEAR	MatrixEQTL modelANOVA
AUPR	0.131679926	0.12847128	0.108051418	0.116587322
RAUPR	1	0.975632993	0.820561052	0.885384173

### The Advantages and Disadvantages of our Method

Compared with the traditional linear regression based eQTL methods [50]–[52], our information-theoretic machine learning method has several advantages: firstly, we use MI to measure the association between genetic marker and gene expression. MI can detect both linear and non-linear dependencies and deal with the heterogeneity of the study population [8], [53]. Thus, our method can identify more eQTLs than can the linear regression models. Secondly, since our method is based machine learning, we use advanced features selection methods - mRMR and IFS, to optimize the affected gene selection. Both mRMR and IFS have been widely used in machine learning areas and many difficult problems have been solved with these feature selection methods [25], [28], [32]–[38]. In mRMR, the maximal relevance guarantees that the selected genes are associated with the genotype of the genetic marker and the minimal redundancy reduces the false positive associations. The IFS method borrows the IFS curve to analyse the performance distribution of a possible affected gene set and determine the optimal affected genes that have the best performance. Both mRMR and IFS methods are easy to understand and practice.

There are still some disadvantages to our method: firstly, since our methods originated from information theory and machine learning, it might be difficult for the traditional statistical geneticist to understand. Some equivalent terms we used may be strange to them, such as the MI we used to measure the association and the IFS curve we used to optimize the affected genes. Secondly, we used the gene expression data to represent the genotype. It is a concept different from the traditional method which is the contrary [7]. Some people may find it difficult to understand. We are of the opinion, however, that the aim of eQTL analysis is to identify the association between genetic marker and gene expression regardless of the representation form taken.

## Supporting Information

Dataset S1 **The outputs of mRMR.eQTL, R/qtl, MatrixEQTL.modelLINEAR and MatrixEQTL.modelANOVA in female and male mice.**
(RAR)

Script S1 **The script of mRMR.eQTL.**
(RAR)
